# Transmission Dynamics and Novel Treatments of High Risk Carbapenem-Resistant *Klebsiella pneumoniae*: The Lens of One Health

**DOI:** 10.3390/ph17091206

**Published:** 2024-09-12

**Authors:** Jiaying Zhu, Taoyu Chen, Yanmin Ju, Jianjun Dai, Xiangkai Zhuge

**Affiliations:** 1 College of Pharmacy, China Pharmaceutical University, Nanjing 211198, China; zhu_jiaying923@163.com (J.Z.);; 2 Department of Nutrition and Food Hygiene, School of Public Health, Nantong University, Nantong 226019, China; 3 Department of Orthopaedics, The First Affiliated Hospital of Baotou Medical College, Inner Mongolia University of Science and Technology, Baotou 014010, China; chty1993@163.com; 4 MOE Joint International Research Laboratory of Animal Health and Food Safety, Key Laboratory of Animal Bacteriology, Ministry of Agriculture, College of Veterinary Medicine, Nanjing Agricultural University, Nanjing 210095, China

**Keywords:** One Health, antibiotic resistance, foodborne, microbiome, phage, nanoparticles, CR-hvKP, KL64

## Abstract

The rise of antibiotic resistance and the dwindling antimicrobial pipeline have emerged as significant threats to public health. The emergence of carbapenem-resistant *Klebsiella pneumoniae* (CRKP) poses a global threat, with limited options available for targeted therapy. The CRKP has experienced various changes and discoveries in recent years regarding its frequency, transmission traits, and mechanisms of resistance. In this comprehensive review, we present an in-depth analysis of the global epidemiology of *K. pneumoniae*, elucidate resistance mechanisms underlying its spread, explore evolutionary dynamics concerning carbapenem-resistant hypervirulent strains as well as KL64 strains of *K. pneumoniae*, and discuss recent therapeutic advancements and effective control strategies while providing insights into future directions. By going through up-to-date reports, we found that the ST11 KL64 CRKP subclone with high risk demonstrated significant potential for expansion and survival benefits, likely due to genetic influences. In addition, it should be noted that phage and nanoparticle treatments still pose significant risks for resistance development; hence, innovative infection prevention and control initiatives rooted in One Health principles are advocated as effective measures against *K. pneumoniae* transmission. In the future, further imperative research is warranted to comprehend bacterial resistance mechanisms by focusing particularly on microbiome studies’ application and implementation of the One Health strategy.

## 1. Introduction

*Klebsiella pneumoniae*, which belongs to the *Enterobacteriaceae* family, can be found extensively in various environments and inconspicuously inhabits the digestive systems of both healthy individuals and animals [1,2]. Additionally, *K. pneumoniae* serves as an opportunistic pathogen responsible for community-acquired and nosocomial infections including urinary tract infections, lower respiratory tract infections, and liver abscesses [3,4,5]. In 1998, *K. pneumoniae* was officially identified as an enteroinvasive foodborne pathogen originating from a contaminated hamburger [6]. This study illuminates a potentially previously unidentified ecological niche for *Klebsiella* that has not been previously recognized as enteroinvasive. Over the past few decades, extensive research has consistently reported the association of *K. pneumoniae* with outbreaks of prevalent foodborne illnesses [7,8,9,10]. Therefore, *K. pneumoniae* is a commensal of animals, causing contamination in meat and vegetables found in supermarkets, and serves as a trigger for extraintestinal infections in humans. 

One Health approaches stem from the understanding that a comprehensive grasp of human, animal, and environmental health cannot be achieved by separately addressing each one. The One Health paradigm originated from the understanding that diseases frequently result from interactions between humans and animals, initially referred to as “one medicine”, and encompassed preventive and public health measures. It has expanded to incorporate environmental science and eco-health, thereby encompassing the collective impact of the environment. The World Health Organization (WHO) and the EU One Health Action Plan emphasize the urgent need to address antimicrobial resistance (AMR) through a comprehensive One Health program. *K. pneumoniae* infections pose significant threats to global health, necessitating the implementation of a “One Health” strategy that recognizes the interconnectedness between human health, animal health, and environmental factors [11]. The concept of One Health has proven effective in various domains such as investigating zoonotic disease outbreaks and assessing biosecurity risks for both humans and animals [12,13]. By adopting a comprehensive One Health approach, concerted efforts have been directed towards devising holistic strategies to mitigate the dissemination and impact of antimicrobial resistance. This involves collaborative interventions, emphasizing proactive measures by global organizations such as the WHO, the Centers for Disease Control and Prevention (CDC), and the Food and Agriculture Organization (FAO).

In the past, *K. pneumoniae* has predominantly caused severe infections in immunocompromised patients; however, more recently, hypervirulent strains have emerged and disseminated, leading to an increase in susceptibility to infections among individuals who are healthy and immunocompetent [14]. Furthermore, the presence of antibiotic-resistant strains of *K. pneumoniae* has significantly complicated the treatment of infections caused by these strains. Recent studies have focused on both antibiotic-resistant and hypervirulent *K. pneumoniae* (HvKP) strains [15,16,17,18]. Livestock has the potential to serve as a reservoir for HvKP, as demonstrated by the isolation of sequence types associated with virulent lineages from raw retail turkey (ST25 [7]) and bovine illnesses (ST65 [19]). The WHO has designated carbapenem-resistant *Klebsiella pneumoniae* (CRKP) as a critical priority pathogen, posing a significant threat to public health. Furthermore, the emergence of carbapenem-resistant hypervirulent *K. pneumoniae* (CR-hvKP) is cause for concern in both disease management and treatment. Therefore, understanding the reservoirs, transmission chains, and epidemiological connections of *K. pneumoniae* is crucial in identifying strategies to reduce the burden of related diseases. 

In this review, we aim to provide a comprehensive analysis of the epidemiology of *K. pneumoniae*, including its resistance and virulence profiles, as well as the key evolutionary pathways of KL64 CRKP and CR-hvKP. Additionally, we will discuss novel antibacterial treatment options and control strategies for these pathogens. Furthermore, we will critically evaluate recent advancements in the field and identify areas that require further attention, while also offering insights into future directions for research on foodborne *K. pneumoniae*.

## 2. The Epidemiology of *Klebsiella pneumoniae* between Environment, Animals, and Humans

Comprehending the potential reservoirs from which pathogenic organisms are acquired is imperative for effectively managing the dissemination of *K. pneumoniae* infection in human populations [20]. *K. pneumoniae* can be found in diverse microbiological niches, encompassing food sources, soil environments, animal dermal surfaces, gastrointestinal tracts, and fecal matter. Notwithstanding its infrequent association with foodborne transmission, investigations into gut colonization preceding *K. pneumoniae* infections lend support to the notion that food may serve as a plausible vector for bacterial transmission [21]. The presence of this common food contaminant in both animal- and plant-based diets is likely a significant contributing factor to the introduction of environmental strains into the human gut, making its discovery in ready-to-eat vegetables particularly concerning [22]. This poses a threat to public health beyond mere food safety concerns. Notably, clinically relevant lineages of *K. pneumoniae* have been identified from distinct non-human sources [23]. Whole-genome data reveal close phylogenetic relationships between *K. pneumoniae* isolates from patients, retail meat [7], and bovine mastitis cases [19]. The 59 bovine isolates were distributed across the species phylogeny, predominantly composed of human isolates, in a study on global diversity [19]. A phylogenetic overlap between isolates from Thai hospitals and those from nearby waterways was identified. Both clinical and environmental isolates exhibited closely related types [24]. Certain populations, including those recognized as global hospital pathogens, demonstrated the ability to transition between habitats and proliferate in multiple environments, facilitating genetic interactions with diverse bacterial species [23].

In addition, animal farming employs antimicrobials specific to animals for growth promotion, disease prevention, and control of *K. pneumoniae* infections. The widespread use of antibiotics in agriculture and livestock production industries has been recognized as a potential cause for the emergence of antibiotic resistance in human healthcare. It is concerning that there are food supply strains of AMR *K. pneumoniae* with high rates found in China [25], South Africa [26], Europe [27], Greece [28], and Iran [10]. The findings suggest that a significant proportion of strains exhibit genetic traits associated with both virulence and resistance, indicating a substantial potential for disease. However, research on food supply resistance remains an underexplored area.

The occurrence of *K. pneumoniae* in the environment can be primarily linked to environmental contamination resulting from sewage spills, inadequate management of hospital waste, and human activities like bathing and swimming, as well as agricultural residue disposal [29]. On the other hand, there is increasing evidence supporting a connection between the hospital setting and transmission of *K. pneumoniae* among patients and healthcare staff members [30,31].

*K. pneumoniae* is believed to have a global presence with significant geographical variation [20]. Recent studies have identified the presence of *K. pneumoniae* in raw hospital sewage worldwide, including in countries such as Thailand, China, England, and Japan [21,24,32,33,34]. Therefore, it is imperative to minimize environmental contamination and subsequent dissemination of *K. pneumoniae* within healthcare facilities. However, limited information exists regarding the persistence and transmission of resistance genes across diverse environmental niches and between microorganisms, animals, and humans.

## 3. Antibiotic Resistance among *Klebsiella pneumoniae*

Exposure to sublethal doses of antibiotics can lead to the emergence of resistance among organisms. The general public is regularly subjected to a range of non-iatrogenic antibacterial substances in their everyday lives, such as the administration of antibiotics in livestock farming for cattle, alongside the excessive utilization of antimicrobial agents in clinical settings. A rise in the frequency of clinically identified drug-resistant bacteria is associated with the increased utilization of antibiotics in both clinical and non-clinical settings. The global dissemination of extended-spectrum β-lactamase (ESBL)-producing *K. pneumoniae* and the subsequent emergence of isolates resistant to colistin and carbapenem medications are the primary drivers behind this resistance. The WHO designates carbapenem-resistant *Enterobacterales* as one of the pathogens with utmost priority. The emergence of ST11 CRKP in the Americas in 1996 marked its subsequent global dissemination, with prominent clones expanding from China’s southeastern coast to inland regions by 2010 [35]. In 2005, a case report described an 80-year-old man with prostate cancer and metastases as the first documented French case of CRKP [36]. This pathogen exhibits a significant fatality rate [20] and has been steadily disseminating worldwide. Of particular concern is the escalating prevalence of CRKP strains in Asia, which poses challenging clinical management dilemmas [37]. 

**Figure 1:**
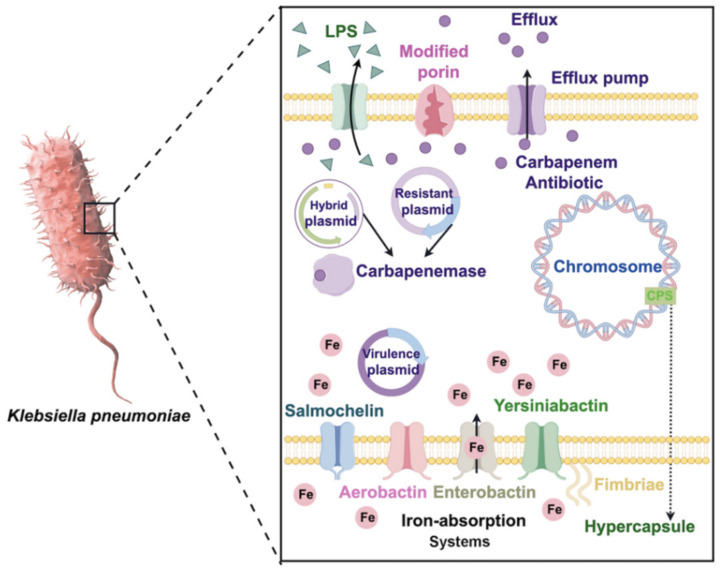
Carbapenem-resistance mechanisms and virulence features of CRKP.

Multiple mechanisms contribute to the emergence of CRKP (Figure 1). These include the production of carbapenemases; overexpression of ESBL and AmpC cephalosporinases along with the reduced expression or deletion of outer membrane proteins; and the activation of efflux pumps (Figure 1). It is noteworthy that the primary mechanism involves producing Ambler class A enzymes such as *Klebsiella pneumoniae* carbapenemase (KPC); class B metalloenzymes like New Delhi metallo-beta-lactamase (NDM), Verona integron-encoded metallo-beta-lactamase (VIM), and imipenemase (IMP); and finally, class D known as oxacillin enzymes (OXA). In 1996, a hospital in North Carolina identified the initial documented strain of *bla*_KPC_ *K. pneumoniae* [38]. The plasmid facilitates transmission of the *bla*_KPC_ coding gene from *K. pneumoniae* strains to other strains through the Tn3-based transposon Tn4401 [39]. 

The other commonly observed gene, NDM-1, was classified as a “superbug” due to its extensive resistance against nearly all antibiotics available during that period [40]. NDM-positive *K. pneumoniae* strains have been detected worldwide [15,41], with the highest prevalence rates reported in the Indian peninsula, the Middle East, and the Balkans [42]. The presence of *bla*_NDM_ has been detected in hospital waste from various countries, including China [43], Europe [44], and Ireland [45], as well as in clinical samples, suggesting the potential utility of sewage monitoring as a cost-effective complement to conventional clinical surveillance. Plasmids, particularly those belonging to the IncX3 subtype, play a pivotal role in facilitating the widespread dissemination of *bla*_NDM_ genes [46]. OXA-48, an Ambler class D carbapenemase, stands out as the most prevalent OXA-48-like enzyme worldwide [47]. Its initial discovery dates back to 2004 when it was isolated from a clinical specimen in Istanbul, Turkey [48]. OXA-48 exhibits hydrolytic activity against penicillins, cephalothin, and imipenem. The composite transposon Tn1999 and its variants harbored on pOXA-48a-like IncL conjugative plasmids are primarily accountable for the current interspecies and global transmission of *bla*_OXA-48_ [49].

Additionally, there has been an upsurge in the emergence of diverse carbapenemases among CRKP strains [50]. These instances demonstrate the potential dissemination of *K. pneumoniae* across various geographical regions [51]. Horizontal transmission facilitates the widespread and facile spread of plasmids carrying these resistance genes among different bacterial species (Figure 1). The presence of *K. pneumoniae* harboring these resistance genes has been detected in humans, in animals (including companion, farm, and agricultural animals, as well as wildlife), and in numerous environmental samples from around the globe [40,47].

Understanding the development and spread of *K. pneumoniae* is crucial for guiding public health measures aimed at reducing the transmission of antibiotic resistance. A crucial step in this procedure involves measuring the impact of foodborne *K. pneumoniae* on the prevalence of antimicrobial-resistant infections in humans. The prospective menace posed by *K. pneumoniae* should be disseminated throughout the entire medical community, particularly those with a vested interest in antibiotic resistance.

## 4. Evolution of KL 64 Carbapenem-Resistant *Klebsiella pneumoniae* in China

As a potential alternative to antibiotics, vaccines targeting surface polysaccharide antigens such as lipopolysaccharide (LPS, O antigen) or capsular polysaccharide (CPS, K antigen) have been proposed for combating multidrug-resistant (MDR) organisms [50,52]. Although experimental animal models have shown the protective efficacy of vaccines and monoclonal antibodies against *K. pneumoniae* [52,53,54], their translation into clinical practice for human use is yet to progress beyond ongoing clinical trials [55]. Furthermore, the extensive diversity in both genetic and phenotypic characteristics among the 79 capsular types (K types) of *K. pneumoniae* presents a significant challenge to achieving comprehensive vaccination coverage [50,56]. According to longitudinal multi-center research conducted in China, there has been a decline in the prevalence of KL47 CRKP across 40 hospitals between 2016 and 2020 [56]. Conversely, KL64 CRKP has exhibited a significant increase from 35.0% in 2016 to 59.6% in 2020, while KL47 decreased from 42.3% to 17.8% during the same period [56]. Similar findings have been reported by other studies as well [50,57,58,59,60,61]. The prevalence of KL64 serotypes significantly increased from 1.54% to 46.08% among ST11 CRKP isolates between 2011 and 2021 [35]. All of these studies consistently reported a gradual subclonal substitution within the dominant clone ST11, with O2v1:KL64 progressively replacing the previously prevalent subclone OL101:KL47 over time in China. Notably, sepsis was found to be more frequently associated with ST11-KL64 CRKP and emerged as an independent risk factor for mortality [50,62]. The predominant ST11 CRKP clones in China are undergoing rapid evolution, giving rise to a high-risk subtype characterized by enhanced pathogenicity and transmissibility. This poses significant challenges for clinical diagnosis, treatment, and infection control within medical institutions [35].

However, various studies present divergent findings regarding the factors contributing to the epidemic success of KL64. One study revealed that O2v1:KL64 exhibited a higher load of mobile genetic elements and a specific point mutation in *recC*, which significantly enhanced recombination proficiency [61]. Conversely, another study proposed that the acquisition of a pK2044-like virulence plasmid from ST23-KL1 hypervirulent *K. pneumoniae* by the sub-lineage ST11-KL64 CRKP conferred an evolutionary advantage by eliminating certain regions associated with increased survival in ST11-KL64 [60]. Nevertheless, both studies acknowledged the association between a hypervirulent sublineage and KL64’s epidemic success. The study conducted by Wang et al. demonstrated that ST11-KL64 represents a genetically diverse lineage consisting of two major clades and a singleton, each originating from different countries and emerging at distinct time points [57]. Chen et al. showed the recombination-driven evolution of CRKP ST11 KL47 to KL64 in China [58]. These findings not only expand our comprehension of the molecular evolutionary history of ST11, but also represent a significant stride towards the development of preventive, diagnostic, therapeutic, and control approaches for CRKP.

Therefore, to efficiently inform and guide vaccine progress strategies aimed at enhancing targeted control, public health efforts should prioritize genomic surveillance to gain the most recent epidemiological insights into the prevailing and clinically significant serotypes, such as K or O antigens.

## 5. Evolution of Carbapenem-Resistant Hypervirulent *Klebsiella pneumoniae*

Infections caused by HvKP can occur in immunocompetent individuals, often in public settings. Initially recognized as a distinct clinical pathogen in Taiwan during the 1980s [37], HvKP has demonstrated higher virulence compared to classical *K. pneumoniae* (cKP), as evidenced by animal lethality experiments involving mice and wax moth larvae, neutrophil assays, and other methodologies. HvKP has disseminated globally from the Asian Pacific Rim, giving rise to invasive diseases such as meningitis, endophthalmitis, and pyogenic liver abscesses [63]. 

Over the past decade, *K. pneumoniae* has rapidly evolved, giving rise to strains that exhibit both multidrug resistance and hypervirulence characteristics simultaneously. The investigation of CR-hvKP has gained significant attention following the 2017 ST11 CR-hvKP epidemic in China [62]. CR-hvKP cases have been reported across continents including Asia [50], Europe, Africa, and America [64]. The genetic origins of CR-hvKP isolates are highly diverse, as are their profiles of antimicrobial resistance. The emergence of antimicrobial resistance traits in HvKP and the acquisition of a hybrid plasmid containing both carbapenem resistance and hypervirulence by *K. pneumoniae* are two prevailing hypotheses for the origin of CR-hvKP [65]. Genes associated with acquired resistance and virulence, which are disseminated among bacteria through mobile genetic elements, include those present in mobilizable and conjugative plasmids, integrons, insertion sequences, and transposons [66,67,68].

Several studies have elucidated the mechanisms underlying the persistent virulence and MDR plasmid evolution in *K. pneumoniae* (Figure 1). The non-conjugative virulence plasmid was capable of transmission with the assistance of a conjugative helper plasmid or through fusion with a conjugative MDR plasmid [69]. Another study reported that the p15WZ-82_Vir plasmid evolved as a result of integration between the hypervirulent plasmid and the MDR plasmid [70]. Furthermore, it has been demonstrated that hybrid conjugative virulence plasmids can readily facilitate conjugation from an ST11 CRKP strain to a CR-hvKP strain [62,71]. Strains exhibiting chromosomal integration of genetic components, including virulence-related features, have also been documented [72]. Furthermore, distinct strains employ diverse mechanisms in the process of horizontal gene transfer (HGT) through which HvKp acquires resistance genes [73]. The most frequently reported mechanisms involve the acquisition of resistance plasmids and resistance genes (Figure 1). Another mechanism involves the accumulation of mutations. For instance, an HvKP strain has developed tigecycline resistance due to the simultaneous overexpression of *AcrAB* and *OqxAB* along with the up-regulation of *RamA* or *RarA*, respectively [74]. Additionally, outbreaks caused by CR-hvKP have been observed in China, indicating that the ability of the host strain to transmit the disease remains unaffected by the presence of additional resistance genes or a plasmid encoding for resistance (Figure 1) [50].

The diagnosis and description of HvKP remain contentious topics to this day [60,75]. Despite the absence of completely accurate and sensitive markers, CR-hvKP has been identified based on various phenotypic and clinical characteristics. Previously, the “String test” was employed for HvKP identification; however, recent studies have demonstrated reduced sensitivity and specificity [76,77]. The virulence genes may offer the most valuable assistance in marker selection. Within the virulence plasmids, *iroB*, *iucA*, *peg-344*, *rmpA*, and *rmpA2* serve as biomarkers for identifying HvKP [78]. HvKP strains can persist within the host and exploit the potential for metastatic dissemination from circulation through the *rmpA*- or *rmpA2*-mediated overproduction of capsule. This mechanism enhances bactericidal activity against complement and inhibits phagocytosis [63]. 

Meanwhile, the HvKP virulence plasmid encodes a metabolite transporter called *peg-344*. It is important to clarify the precise role of *peg-344*. Additionally, *iucA* encodes aerobactin and may be considered a stable genetic marker [78]. The recent emergence of ST11 CR-hvKP strains in China has garnered global attention due to the discovery of a pLVPK-like virulence plasmid. This plasmid has the potential to cause severe infections in individuals who are otherwise in good health, posing challenges for treatment using existing therapies [71]. High levels of pathogenicity and resistance often result in unfavorable therapeutic outcomes. The clinical criteria present particular challenges, as the patient’s profile is influenced by their immunological health, underlying illnesses, and the specific type of infection [5]. Consequently, comparing data across different studies becomes challenging due to the potential inconsistency in meeting the criteria for identifying HvKp strains between studies [3,60,75].

To determine the virulence of CR-hvKP, a variety of tests including the string test, the human neutrophil killing assay, and the *Galleria mellonella* model have been employed by most studies. However, a globally accepted definition for CR-hvKp is still lacking. A comprehensive understanding of the molecular evolutionary mechanisms underlying virulence- and resistance-bearing plasmids, as well as the transmission characteristics of these clones, could facilitate the improved tracking and management of these organisms. Greater efforts are required to eradicate or impede the development of CR-hvKP and hv-CRKP strains. Urgent action must be taken to mitigate the incidence and impact posed by this potentially fatal pathogen.

## 6. The Treatment of *Klebsiella pneumoniae*

Bacterial cultures and subsequent antibiotic susceptibility testing are commonly employed to establish the optimal antibiotic treatment regimen for *K. pneumoniae* infections. β-lactams (including penicillins and carbapenems), β-lactamase inhibitors (such as piperacillin-tazobactam), polymyxins (colistin or polymyxin B), cephalosporins (cefepime), or tigecycline represent frequently utilized therapeutic options for managing *K. pneumoniae* infections (Table 1). Given the restricted range of available therapies and historically sluggish progress in identifying new antimicrobial drugs, infections caused by carbapenem-resistant *Enterobacteriaceae* (CRE) have become a major worldwide public health issue. Given the high fatality rate associated with CRKP, early detection and intervention are crucial in mitigating the severity of illness and reducing mortality. Current management strategies for CRKP are primarily based on clinical guidelines. Combining traditional medications such as aminoglycosides, aztreonam, polymyxins, and tigecycline with other therapeutic agents has demonstrated enhanced efficacy in the treatment of severe CRKP infections (Table 1). However, the options for CRKP therapy remain limited. Currently, ceftazidime-avibactam, meropenem-vaborbactam, and imipenem-relebactam carbapenems are considered novel first-line antibiotics for managing CRKP infection diseases [79]. Importantly, therapeutic medicine should be tailored to specific circumstances due to the diverse epidemiological situations and strain enzymes in different regions. Each emerging resistance mechanism can be gradually addressed through novel derivatives of currently available antibiotics [80]. Moreover, deep learning techniques hold great potential for discovering structurally unique antibacterial compounds, thereby expanding our arsenal of antibiotics [81]. To minimize the time gap between antibiotic introduction and resistance development, it is crucial to develop antibiotics targeting new bacterial targets.

### 6.1. Aztreonam 

In the case of Class B and D carbapenemases strains, aztreonam demonstrates efficacy; however, its effectiveness is compromised in strains that produce ESBL genes capable of hydrolyzing aztreonam. Combining aztreonam with ceftazidime-avibactam may potentially offer an effective treatment option [79,82]. Significantly, the combination of aztreonam-avibactam effectively inhibited all MBLs isolates examined in a study [83].

### 6.2. Polymyxins

The polymyxin antibiotics colistin and polymyxin B are employed for the treatment of CRKP infections. However, in recent years, CRKP strains have developed resistance to these drugs. Polymyxins specifically target lipid A, a constituent of the lipopolysaccharide component of the outer membrane. Changes in lipid A and several *mcr* genes [84] contribute to polymyxin resistance. The *mcr*-carrying plasmids are found in *K. pneumoniae* isolated from human, animal, food, and environmental samples.

Additionally, detecting colistin resistance in vitro can be challenging due to heteroresistance arising from minor resistant subpopulations, which subsequently leads to treatment failure. The data collected from the European Antimicrobial Resistance Surveillance Network (EARS-Net) reveal that in countries with a high prevalence of CRKP, such as Greece and Italy, there is a notable increase in resistance rates to polymyxins among CRKP strains compared to those susceptible to carbapenems. In China, the resistance rates of *K. pneumoniae* to polymyxin B and polymyxin E have witnessed an upward trend, rising from 1.1% and 1.2% in 2018 to 4.8% and 2.8% in 2022, respectively. Similarly, the resistance rate of CRKP to polymyxins has shown an increase from 3.6% in 2020 to 8.2% in 2022 (CHINET data).

Nevertheless, their nephrotoxicity and limited clinical effectiveness impose constraints on their utility [20,85]. In certain regions where it is frequently the only effective antibiotic against CRE infections, polymyxin is regarded as a “highest priority” critical antimicrobial by WHO. 

### 6.3. Tigecycline

One of the most significant last-resort antibiotics for treating infections caused by highly drug-resistant bacteria is tigecycline, particularly in the case of *Klebsiella pneumoniae* (C-C-RKP), which is resistant to both colistin and carbapenem. In recent years, rare instances of tigecycline resistance have been observed, primarily due to chromosome-encoded mechanisms such as overexpression of efflux pumps and ribosome protection [86]. The plasmid-mediated mobile tigecycline resistance gene *tet(X4)* has been reported in China [87]. Additionally, this mobile resistance gene *tet(X4)* can still be found in soils that have received fertilizer and animal dung that has undergone various composting processes [88,89,90]. A high-risk ST16 clone was initially identified during a prevalence investigation into tigecycline resistance in C-C-RKP isolated from Thailand. Their primary mechanism of tigecycline resistance is associated with the overexpression of the regulator *RamA* and the efflux pump gene *acrB* [3]. However, due to its limited tissue penetration and rapid dispersion after intravenous administration, tigecycline exhibits inadequate clinical efficacy against urinary tract infections and primary bloodstream infections [91].

### 6.4. Ceftazidime-Avibactam

Novel combinations of β-lactamase inhibitors have emerged and gained approval, with a specific focus on combating MDR microorganisms like CRE [79]. In 2015, avibactam, the first carbapenemase-resistant β-lactamase inhibitor, was introduced to the market in combination with ceftazidime [92]. Avibactam is a synthetic diazabicyclooctane (DBO) non-lactam compound that exhibits activity against class A (KPC) and class D (OXA-48-like) carbapenemases but not against class B metallo-β-lactamases. It forms reversible covalent bonds with serine β-lactamases including NDM, VIM, and IMP types of metallo-β-lactamases [93]. Ceftazidime-avibactam and aztreonam, however, demonstrate significant in vitro synergy that confers efficacy against MBL isolates. Studies have indicated the superiority of ceftazidime-avibactam over polymyxin antibiotics for treating CRE infections due to its favorable safety profile and absence of toxicities [94,95]. It is currently available on the market. The combination of metronidazole and ceftazidime-avibactam is authorized for the treatment of challenging intra-abdominal infections. Several documented mutations have been found to increase resistance, predominantly in carriers of the KPC-2 and KPC-3 enzymes. According to reports, there are indications of the transfer of KPC-3 onto another plasmid, modifications in the porin channels OmpK35 and OmpK36 [96], and an increase in the expression levels of efflux pumps [97]. Of concern are single amino acid changes at D179Y/T243M, D179Y, and V240G that impact the Ω-loop in KPC-3, resulting in avibactam resistance [98]. Recently, novel variants named KPC-31 and KPC-115 were isolated from ceftazidime-avibactam-resistant *K. pneumoniae* strains belonging to the ST11 lineage in Argentina [99].

### 6.5. Cefiderocol

A siderophore-based cephalosporin, known as cefiderocol, exhibits enhanced stability against serine and metallo-carbapenemases. Cefiderocol exploits the pathogen’s iron uptake requirement at infection sites, acting as a Trojan horse. Moreover, it demonstrates potent activity against *Enterobacteriaceae*, *Acinetobacter baumannii, Pseudomonas aeruginosa*, *Burkholderia cepecia*, and *Stenotrophomonas maltophilia*; thus making it a valuable empirical or targeted treatment option for various infectious diseases [100,101]. However, its anaerobic and Gram-positive efficacy is suboptimal based on clinical data analysis, necessitating adjunctive therapy with other drugs.

We are now witnessing the emergence of some promising β-lactam/β-lactamase inhibitors. Aztreonam/avibactam and three cefepime-based combinations (cefepime/enmetazobactam, cefepime/taniborbactam, and cefepime/zidebactam) are at the late stage of development. In April 2024, aztreonam/avibactam was granted marketing authorization by the European Medicines Agency (EMA) in the European Union (https://www.ema.europa.eu/en/news/new-antibiotic-fight-infections-caused-multidrug-resistant-bacteria; accessed on 6 May 2024). Aztreonam/avibactam exploits the unique property of aztreonam to resist hydrolysis by MBL in combination with the potent activity of avibactam against serine-type enzymes, effectively providing coverage against all types of β-lactamases. In vitro studies have demonstrated that *Enterobacterales* can acquire resistance to cefiderocol by producing both serine and metallo-beta lactamases [102]. Nevertheless, the incorporation of avibactam holds promise in overcoming this form of resistance [103]. These therapies have the potential to make a substantial progress in combating Gram-negative infections.

### 6.6. Nanoparticles 

In recent years, there has been an increase in the utilization of nanoparticles (NPs), which demonstrate broad-spectrum efficacy against pathogenic microorganisms. Compared to conventional antibiotics, nanomaterials facilitate targeted interactions with bacterial cells through deliberate engineering designs encompassing size regulation, surface modifications, crystalloid adjustments, and stimuli-responsive functionalization [104]. Metallic NPs such as Ag, Cu, Au, CeO_2_, ZnO, etc., carbonaceous nanomaterials including GO, graphene and carbon nanotubes, borides like Boron nitride (BN), nanosized polymers such as polycarbonate, and nanocomposites like La_2_O_3_/Ag-GO have been extensively reported as five types of antimicrobial nanomaterials. While other antimicrobial nanomaterials exhibited relatively inferior efficacy compared to antibiotics, Ag NPs, Au clusters, ZnO, and CuO nanomaterials demonstrated robust antibacterial efficiency with minimum inhibitory concentrations comparable to pharmaceutical drugs. Nano–microbe interactions are less understood compared to antibiotic cell internalization mechanisms. The majority of nanomaterials exhibit limited ability to penetrate the cytoplasm, instead primarily engaging with the cell wall and membrane components through processes involving instability, oxidative stress, mechanical disruption, and thermal impacts [105,106,107].

Six pathways exhibiting antimicrobial activity have been identified, including extended ionic death, catalytic killing, membrane destruction, interruption of the electron transport chain, and cell entrapment mediated by nanoparticle aggregation. For example, BN nanosheets were found to possess potent antibacterial action against five resistant strains by arresting cell division [108]. Furthermore, NPs do not contribute to the development of antibiotic resistance [109]. However, recent research has revealed that the evolution of nanomaterials under non-lethal selective pressure can give rise to diverse resistance mechanisms, encompassing mutations in the efflux system [110], suppression of the outer membrane porin family [111], HGT [112,113], flagellin production [84,114], and remodeling of the cell envelope [115]. Moreover, deactivation of nanomaterials can be achieved through ionic settling and nanomaterial gathering. The resulting array of antimicrobial effects stemming from AMR evolution contributes to variations in nano–microbial interactions and antimicrobial mechanisms. The prevention of the evolution of AMR necessitates the specific design and meticulous utilization of antimicrobial nanomaterials, encompassing the engineering of nanocomposites to enhance interactions at the nano–microbe interface, the incorporation of ligands that precisely target bacterial eradication, and the implementation of doping or shielding techniques to avert ion release. To effectively combat AMR superbugs, substantial endeavors should be dedicated to logically advancing antimicrobial nanomaterials.

Nanodrug delivery systems (DDSs) have emerged as innovative therapeutic approaches for life-threatening diseases [116]. DDSs enable enhanced drug retention in the bloodstream, reduce non-specific distribution, and target drug delivery to infection sites. The combination of nanomaterials and antibiotics holds promise for improving the therapeutic index [16]. Numerous studies have utilized Ag, Au, Cu, Fe, Ti, and mesoporous silica nanoparticle-based DDSs for treating infectious diseases [16]. Nanomaterial-based delivery techniques such as pH-triggered, enzyme-dependent, and bacterial toxin-triggered DDSs can achieve spatiotemporal antibiotic delivery. For instance, the synergistic antibacterial activity of S-thanatin and tigecycline enhances the antibacterial efficacy of nanorods against tigecycline-resistant *K. pneumoniae* strains [16]. 

When utilizing nanoparticles in the field of medicine, numerous considerations still need to be considered. Clinical applications continue to face challenges related to limited dispersibility, low selectivity, ambiguous toxicity, entrapment within the mucosal layer and reticuloendothelial system, extracellular matrices of biofilms, and capture by macrophages.

### 6.7. Phage Therapy 

The expanding AMR epidemic has reignited research on alternatives, and bacteriophage treatment stands out as one of the most promising approaches. Coined by scientist Felix d’Herelle in 1917, the term “bacteriophage” originates from the Greek word meaning “bacteria eater” [11]. Bacteriophages possess the ability to infect and propagate within bacteria, making them the oldest and most prevalent creatures on Earth with an estimated abundance of 10^31^ particles in the biosphere [117,118]. Recognizing its potential, phage treatment was included as a method to combat antibiotic resistance by the US National Institute of Allergy and Infectious Diseases in 2014 [119]. In the United States and the European Union, phages are considered pharmaceutical products subject to stringent regulations governing their manufacture and marketing authorization, including adherence to Good Manufacturing Practices (GMP). Moreover, established phage treatment programs currently exist in Belgium, France, and Sweden, as well as in Georgia and Poland where such programs have been implemented for a considerable period [117]. Collaborative efforts within Europe and Australia have effectively facilitated the development of standardized phage treatment regimens for enabling therapeutic applications [120]. The UK has recently announced its intention to commence the evaluation of requests for compassionate-use phage treatment submitted through the National Health Service. These phage treatments have the potential to address AMR resulting from interconnections between humans, animals, and the environment [117].

Phage therapy exhibits superior selectivity in targeting bacteria without disrupting the body’s natural microbiota, having localized reproduction at the infection site, and having potent bactericidal efficacy against antibiotic-resistant bacteria [121,122]. Given its prior authorization for antibacterial applications in agriculture and food processing sectors [123], the resurgence of antibacterial resistance has reignited Western interest in exploring phages’ medicinal potential [94]. As a viable alternative treatment for severely drug-resistant illnesses, phage therapy holds promise [124,125,126]. Encouraging results from in vitro studies have identified numerous phages exhibiting activity against various CRKP isolate strains [127]. Subsequent investigations using murine models have demonstrated the successful treatment of *K. pneumoniae* infections with phages [128]. Notably, mouse survival rates were found to be more influenced by the timing of phage delivery rather than by dosage, as revealed in certain mouse experiments [129,130]. A combination therapy involving a cocktail of *K. pneumoniae* phages effectively eradicated pathobionts from the murine gut microbiota [128]. In a Phase 1 trial, the safety and persistence of therapeutic phages were convincingly demonstrated, providing promising avenues for further research on CRKP-associated infections. The potential application of this strategy in clinical practice, particularly for persistent infections, is demonstrated by recent case reports on the utilization of phages against *K. pneumoniae* [131]. A study conducted by Qin et al. presented a case where a MDR strain of *K. pneumoniae* caused a multifocal urinary tract infection that was effectively treated with a phage cocktail [132]. Furthermore, an instance involving oral and intra-rectal administration of phages against KPC-producing CRKP was described [131]. 

Phage treatment represents an additional precision medicine strategy, harnessing the unique ability of phages to selectively modulate the gut microbiota and target specific sites for medication delivery. Phages naturally occur as bacterial predators that inhabit diverse ecosystems. By leveraging advanced phage engineering tools, drugs can be attached to phage surfaces and released upon reaching their intended targets [133]. This approach enables selective administration of high dosages at specific locations, thereby minimizing systemic drug exposure and reducing off-target tissue damage [122]. The potential widespread adoption of phage-mediated medication delivery holds significant promise in clinical practice.

Apart from that, biological challenges associated with phage therapy include the following: (a) the risk of selecting phage-resistant microbes, (b) complexities in pharmacodynamics and pharmacokinetics, and (c) interactions between the phages and human hosts (such as phage-specific immunity) [134]. Therefore, when developing a therapeutic phage cocktail, it is crucial to consider factors such as host range, receptors, and infectious efficacy. Several potential solutions to these issues have been proposed including modifying therapeutic phages [118,135], employing combination therapy [128,136], conducting rigorous clinical trials [137], genome modification techniques [138,139,140,141,142,143,144], and utilizing synthetic phage genomics approaches [145]. In addition, the establishment of phage libraries, prepared in accordance with GMP standards, along with optimized screening techniques, will contribute to the advancement of phages as a contemporary form of medicine. These advancements have the potential to make significant strides in the management of nosocomial infections.

In general, innovative therapeutic strategies have the potential to effectively combat life-threatening infections and enhance the longevity of medications. Several countries including the United States [146], Belgium, France, Sweden, Australia, and the United Kingdom have implemented a “parallel track” approach allowing compassionate use of phage therapy on an individual basis when conventional antibiotic treatments fail, even in the absence of clinical trial evidence supporting its efficacy. To facilitate further progress in this field, it is imperative to conduct more comprehensive fundamental and preclinical research as well as meticulously planned randomized double-blind placebo-controlled trials.

### 6.8. Antimicrobial Peptides

The emergence of CRKP against existing antibiotics poses a significant concern for humanity, emphasizing the urgent need for novel treatments with antibacterial properties and biosafety. The utilization of antimicrobial peptides (AMPs) has shown promising potential as an effective alternative in addressing resistance against this bacterium. The presence of these peptides has been widely observed across various organisms, with a typical length of fewer than 60 residues. They commonly exhibit positive charges ranging from +1 to +5 and can be categorized as amphipathic compounds [147]. The reorganization of peptides and proteins, an inherent and modifiable phenomenon, provides diverse avenues for the fabrication of functional nanomaterials with antibacterial properties in the field of biological science. In particular, the recognition of supramolecular nanomaterials based on short peptides has witnessed a significant surge owing to their facile fabrication process, advantageous physicochemical characteristics, and diverse array of structural functionalities [148]. 

The bactericidal effects of AMPs can be attributed to both membrane lysis and non-membrane lysis mechanisms [149]. The AMPs possessing membranolytic properties interact with outer membrane structures and lipids, resulting in the disruption of membranes. Conversely, non-membranolytic AMPs specifically target intracellular components such as DNA, RNA, and proteins to disrupt bacterial cell function [148]. By modifying arenicin-3, the positive charge is enhanced while reducing its lipophilicity. Consequently, AA139 has been developed and is currently undergoing preclinical trials. This peptide has exhibited remarkable efficacy against multidrug-resistant *K. pneumoniae* strains, with significantly reduced cytotoxicity and hemolytic effects on human cells [150]. The speed of antibiotic discovery has been significantly improved through the recent development of novel computational methods, which include the identification of AMPs [151,152]. Santos-Júnior et al. have employed a machine learning technique on an extensive collection of 63,410 publicly accessible metagenomes from diverse regions worldwide, in addition to 87,920 genomes of bacteria and archaea, to proactively identify and classify potential antimicrobial peptides (c_AMPs). The development of AMPSphere, a freely accessible and openly available database containing 863,498 unique peptides and 6499 families of antimicrobial peptides from 72 diverse environments, has facilitated the identification of approximately one million novel antibiotics within the worldwide microbiome using advanced machine learning techniques [152]. 

Despite the promising antimicrobial efficacy demonstrated by various AMPs in both laboratory and animal studies, their potential as therapeutic agents faces multiple obstacles. One crucial aspect impacting their suitability as drugs is their susceptibility to degradation within the physiological environment, which significantly influences the pharmacokinetics and bioavailability of AMPs [153]. The potential impact of AMPs’ broad-spectrum antimicrobial properties [154] should also be taken into consideration, as they may disrupt the natural microbiota in a manner similar to that observed with antibiotic use. Challenges in utilizing AMPs as therapeutic agents encompass limited information regarding their safety profile, discrepancies between in vivo and in vitro activity, and the substantial expenses associated with manufacturing without commercially viable platforms for AMP production [155]. In addition, there have been reports indicating the emergence of resistance to AMPs [156]. Bacterial pathogens have developed defensive mechanisms, primarily involving modifications in their outer envelope through the production of capsules and alterations in lipopolysaccharides. Additionally, they employ strategies such as sequestration and degradation of AMPs to evade host defenses.

Regarding these matters, recent advancements have highlighted various approaches and tactics, including the utilization of nanoformulations for delivery systems. These developments offer promising opportunities for their practical application in the upcoming years [151,157]. Similarly, further investigation into the potential synergistic effects of AMPs and antibiotics may provide an alternative approach for harnessing AMPs in the treatment of *Klebsiella pneumoniae* infections. However, additional research is necessary to comprehensively explore the behavior of AMPs as a novel therapeutic option.

## 7. Vaccine

It is imperative to expedite the development of a vaccine specifically targeting *K. pneumoniae*. Various vaccination strategies can be explored, including whole organism vaccines, capsular polysaccharide-based vaccines [55], lipid polysaccharide-based vaccines [158], recombinant or cultured protein-based vaccines [159], extracellular vesicle-based vaccines [160], ribosomal-based immunizations [161], genetic vaccines [162], and multi-epitope vaccines [163]. Currently, there are two primary target groups for *K. pneumoniae* vaccines, each with distinct delivery approaches. The initial group consists of expectant mothers in their second or third trimester of pregnancy, aiming to enhance the transfer of protective antibodies through the placenta and safeguard newborns during their vulnerable neonatal period and early months of life. The second approach aims to cater to individuals who are more vulnerable to *K. pneumoniae* infection, including pediatric, adolescent, and adult populations at increased risk due to factors such as prolonged hospitalization or residence in long-term acute care facilities. This group also encompasses individuals with chronic obstructive airway disease, a heightened susceptibility to surgical site infections and device-related infections, compromised immune systems, or hematological and other malignancies.

The burden could be alleviated by an efficient vaccine through various mechanisms. An effective immunization would reduce this load via multiple pathways [164]. Furthermore, by reducing infection rates, vaccines have the potential to mitigate the need for employing more potent antibiotics. Consequently, this would alleviate selective pressure on *K. pneumoniae* and potentially delay the emergence of AMR. The safety, efficacy, and production feasibility of vaccines still face obstacles despite the progress made in this field. The bacterium presents formidable challenges due to its intricate phenotypic and genotypic characteristics. Furthermore, the lack of suitable animal models poses a significant obstacle that could potentially impede progress in vaccine development for Klebsiella strains [165]. As the field advances, utilizing computational techniques and AI has significant potential to revolutionize the process of developing vaccines targeting *K. pneumoniae*, thereby facilitating effective approaches in combating this medically important pathogen.

## 8. Infection Control

In addition to the development of antimicrobial drugs, the ultimate efficacy in combating *K. pneumoniae* relies on appropriate infection control methods. Moreover, prompt treatment of *K. pneumoniae* infections is crucial due to their hypervirulent nature and potential for metastatic spread, which can cause significant damage to vital organs. Within healthcare facilities, there remains a pressing need for enhanced awareness and adherence to infection control measures.

The management of infections necessitates the implementation of a comprehensive and coordinated plan, as well as strict adherence to infection prevention guidelines. Key control strategies encompass source control, transmission prevention, host defense, and protection of vulnerable populations. To effectively curb the dissemination of *K. pneumoniae* from its origin, extensive screening, identification efforts, educational initiatives, and multimodal interventions are imperative. Essentially, healthcare professionals should prioritize the avoidance of unnecessary invasive equipment such as indwelling urinary catheters or intravenous infusion lines, maintain strict hand hygiene practices, reinforce barrier measures, and implement isolation protocols for patients colonized or infected with carbapenemase producers [166]. Furthermore, addressing the main concerns regarding medical equipment cleaning and environmental colonization requires active surveillance of CRE carriers through early rectal screening upon hospital admission, rigorous environmental cleaning procedures, case tagging, and contact tracing to effectively prevent *K. pneumoniae* transmission and outbreaks [15]. Additionally, it is crucial to enhance antimicrobial stewardship efforts in order to preserve the long-term effectiveness of medications [20,51].

Further research is imperative to investigate the impact of *K. pneumoniae* infections on clinical medicine and public health, considering the globalized food commerce, extensive food distribution networks, and intensified food production.

To mitigate the transmission of *K. pneumoniae* across environmental, animal, and human sectors, we propose enhanced education regarding antimicrobial stewardship, intensified monitoring and detection of *K. pneumoniae* in veterinary microbiology, comprehensive surveillance of their epidemiology in both humans and avian species, strict adherence to hand hygiene protocols at slaughter facilities as well as milking facilities, and rigorous control measures to minimize contamination levels on meat products. Moreover, incorporating information on antibiotic usage in animals would significantly impact policies related to antimicrobial stewardship across all domains. Recognizing and addressing the zoonotic threat posed by *K. pneumoniae* would substantially enhance food safety standards and promote human health.

Human activities have the greatest direct impact on surface waterways utilized for leisure, drinking water, animal, and vegetable feeding. However, natural waterways, sediments, soils, and periphyton present challenges for the storage and spread of *K. pneumoniae* and antibiotic resistance genes to natural bacteria. Few randomized controlled trials are investigating therapies targeting the aqueous environment to lower *K. pneumoniae* infection rates. Nevertheless, measures such as reducing overall antibiotic usage, using antibiotic alternatives (e.g., bacteriophages), and promoting education in agriculture for prudent antibiotic practice could prevent environmental transmission. Additionally, monitoring *K. pneumoniae* in various environmental matrices including aerosol, periphyton, and indoor surfaces is necessary. Similarly, interventions for cleaning and disinfecting aquatic environments include routine acetic acid cleaning techniques; chemical or temperature-controlled water sterilization; and removal of impacted plumbing systems.

In summary, optimizing the One Health approach would entail establishing standardized protocols for evaluating antimicrobial susceptibility in environmental and animal samples, alongside human samples. Furthermore, formulating comprehensive policies regarding antibiotic usage in both medical and agricultural domains is crucial. Taking proactive measures to reduce the prevalence of potential infections, mitigate their impact, and minimize their transmission to humans and the environment are of paramount importance.

## 9. Co-Occurrence Analysis

The bibliometric study was conducted using the Web of Science (WoS) Core Collection and PubMed, which are widely regarded as the premier database for bibliometric analysis [167,168]. A search query was formulated to extract data from WoS and PubMed with the following criteria: (Theme = *Klebsiella pneumoniae*) and (Theme = foodborne or animals or avian or livestock or poultry or food) and (Language = English) and (Document types = Article) and (Published year = 2003–2023). A total of 2404 articles were included in the analysis.

**Figure 2:**
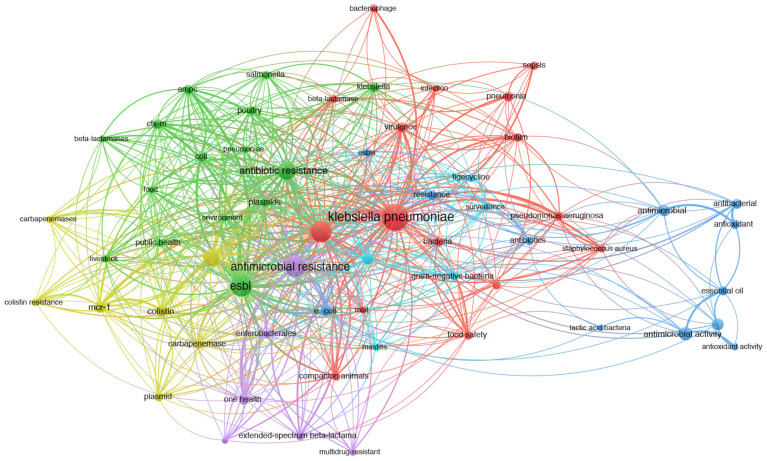
Visualization depicting the keywords related to *Klebsiella pneumoniae*. The varying sizes of the data points correspond to their respective frequencies.

**Figure 3:**
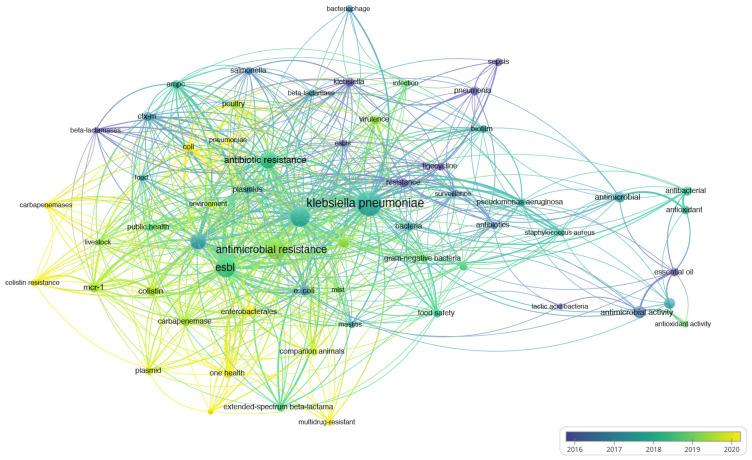
Keywords distributed based on order of appearance over time. Blue keywords precede yellow ones.

**Figure 4:**
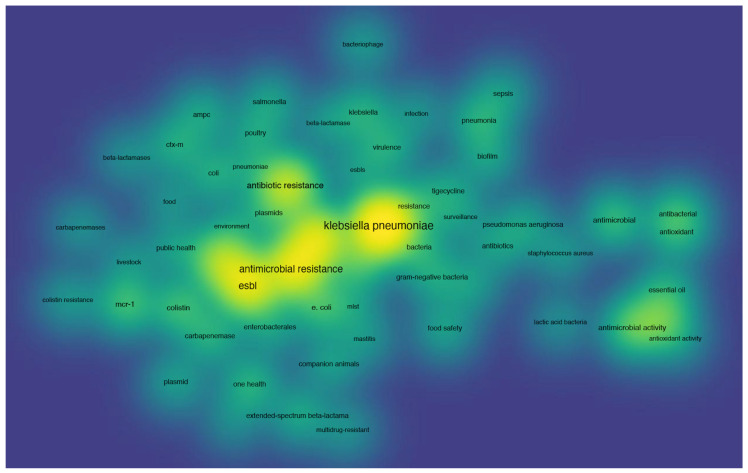
The keywords categorized based on frequency of occurrence. The ones highlighted in yellow are the most frequent and those in green follow closely behind.

Co-occurrence analysis utilizes the frequency of co-appearance of two items in publications to establish their association [167,168]. The final analysis encompasses keywords provided by the authors and occurring more than fifteen times in the WOS core database. A total of 61 keywords were identified, roughly categorized into three clusters: “antimicrobial resistance”, “one health”, and “genetic study” (Figure 2). The color-coded representation of publication keywords reflects their average occurrence over time (Figure 3), with current publications depicted from blue to yellow. Notably, a significant number of studies have focused on antimicrobial resistance, including carbapenemases, colistin resistance, and extended-spectrum beta-lactamases (Figure 4). The emergence of colistin resistance has led to increased attention towards “One Health” and “the microbiome”, which will continue to be crucial areas for future research. Hereafter, we outline several important directions for further investigation based on our perspective.

- **Colistin resistance.** Given the indispensability of polymyxins in human therapy, the WHO accords them the utmost priority among antibiotics for curbing animal usage. The implementation of a colistin withdrawal strategy and subsequent decline in its agricultural application have significantly contributed to mitigating colistin resistance in both humans and animals. Despite limited knowledge on this matter, the persistence of *mcr-1* and colistin resistance suggests that factors beyond animal colistin usage may influence CRKP epidemiology. Therefore, continuous monitoring of colistin is imperative to serve as an alert system for promoting prudent use in hospitals. Furthermore, the rational design of nano-antibiotics and phages emerges as a promising approach to combat the crisis posed by colistin resistance.
- **One health.** The concept of One Health is exemplified by the global impact of antibiotic usage in animals, highlighting the imperative for enhanced collaboration across environmental, animal (domestic and wildlife), and human health sectors. However, similar power imbalances and inefficiencies that persist in global health governance also influence the governance of One Health. To facilitate informed decision-making ensuring both food security and public health protection, integrated approaches are required to elucidate the relationship between antibiotic use in animals and the burden of antibiotic resistance in humans. We advocate for novel infection prevention and control programs, founded on the pillars of One Health, to reduce Gram-positive hospital-associated pathogen transmission.
- **Microbiome.** Technological advancements such as next-generation sequencing [50,61], RNA sequencing [60], bacterial droplet-based single-cell RNA-seq [169], and deep learning approaches [81] have enhanced our proficiency in identifying and investigating antibiotic resistance. These emerging technologies and methodologies complement established culture-based methods, enabling rapid and accurate determination of resistance in both cultivable and non-cultivable bacteria for clinical and surveillance applications. Enhanced in vitro sequencing methodologies and computational algorithms for genomic data organization and antibiotic resistance prediction are being employed to address the specific challenges associated with comprehending and evaluating genetic determinants of resistance using sequencing data. However, ensuring the accuracy of reference databases is imperative for all subsequent analyses. Therefore, the systematic annotation of recently identified genes and the avoidance of misinterpretations will greatly benefit both fundamental science and public health.

## 10. Conclusions and Outlook 

The global expansion of *K. pneumoniae* poses a significant threat to public health and the efficacy of antibiotics, with potential dissemination across human, animal, and environmental domains having severe implications for both human and animal well-being. The escalating prevalence of ST11 hv-CRKP presents a substantial public health concern, further exacerbated by the mobility of virulence-associated plasmids. Future clinical practices may encounter substantial challenges due to the exceptional virulence and drug resistance exhibited by CR-hvKP on a global scale. Prospective studies should prioritize investigating the integration of the virulence plasmid into the chromosome of CR-hvKP as it holds promise in unraveling molecular mechanisms underlying CR-hvKP. Additionally, it is advisable to take into account the potential utilization of KL47 and KL64 capsule variants in forthcoming vaccine research. Furthermore, while phage therapy and nanoparticles offer potential alternative treatments for drug-resistant illnesses, their clinical applicability remains uncertain. Therefore, interdisciplinary collaboration is essential to address the complexities of infection management. Prospective studies employing the One Health approach and integrating epidemiological data collected from human, animal, and environmental sectors would generate valuable surveillance data, enhancing our comprehension of the cross-sector transmission of *K. pneumoniae*. Population genomic analysis is imperative to investigate the reservoirs contributing to the amplification and dissemination of AMR and virulence determinants, as well as to identify potential pathways for interrupting transmission to humans in disease control efforts.

## Figures and Tables

**Table 1:** Spectrum of activity of anti-carbapenem-resistant *Klebsiella pneumoniae* therapeutics.

Agent	Class A	Class B	Class D	Mechanisms
Aztreonam	−	+	+	Inhibits bacterial cell wall synthesis
Fosfomcymin	+	+	+	Inhibits bacterial cell wall synthesis
Colistin, Polymyxin B	+/−	+/−	+/−	Destruction of bacterial cell membrane
Tigecycline	+/−	+/−	+/−	Inhibits protein synthesis
Ceftazidime- avibactam	+	-	+	β-lactam-β-lactamase inhibitor combinations
Meropenem- vaborbactam	+	-	-	β-lactam-β-lactamase inhibitor combinations
Imipenem- relebactam	+	-	-	Non-β-lactam bicyclic DBO β-lactamase inhibitor
Cefiderocol	+	+	+	“Trojan horse” straegy
Plazomicin	+	+	+	Inhibits protein synthesis
Nacubactam	+	+/−	+	β-lactamase inhibitor
Zidebactam	+	+/−	+	β-lactamase inhibitor
Taniborbactam	+	+	+	β-lactamase inhibitor
Phage therapy	+	+	+	Use of host machinery for their replication
Nanoparticles	+	+	+	Destruction of bacterial cell membrane; disruption electron transport chain; catalytic killing; ionic killing; cell division arrest
Antimicrobial peptide	+	+	+	Interacts with LPS and/or phospholipids /DNA/capsule/protein
Vaccine	+	+	+	Humoral response

## Data Availability

All data generated or analyzed are included in this review. Graphic abstract and Figure 1 are drawn by Figdraw 2.0.
